# The Potential Role of Polyphenols in Oxidative Stress and Inflammation Induced by Gut Microbiota in Alzheimer’s Disease

**DOI:** 10.3390/antiox10091370

**Published:** 2021-08-27

**Authors:** Umair Shabbir, Akanksha Tyagi, Fazle Elahi, Simon Okomo Aloo, Deog-Hwan Oh

**Affiliations:** Department of Food Science and Biotechnology, College of Agriculture and Life Sciences, Kangwon National University, Chuncheon 200-701, Korea; umair336@gmail.com (U.S.); akanksha.tyagi001@gmail.com (A.T.); elahidr@gmail.com (F.E.); okomosimon@gmail.com (S.O.A.)

**Keywords:** antioxidants, anti-inflammation, neurodegeneration, microbiota-gut-brain axis, gut dysbiosis

## Abstract

Gut microbiota (GM) play a role in the metabolic health, gut eubiosis, nutrition, and physiology of humans. They are also involved in the regulation of inflammation, oxidative stress, immune responses, central and peripheral neurotransmission. Aging and unhealthy dietary patterns, along with oxidative and inflammatory responses due to gut dysbiosis, can lead to the pathogenesis of neurodegenerative diseases, especially Alzheimer’s disease (AD). Although the exact mechanism between AD and GM dysbiosis is still unknown, recent studies claim that secretions from the gut can enhance hallmarks of AD by disturbing the intestinal permeability and blood–brain barrier via the microbiota–gut–brain axis. Dietary polyphenols are the secondary metabolites of plants that possess anti-oxidative and anti-inflammatory properties and can ameliorate gut dysbiosis by enhancing the abundance of beneficial bacteria. Thus, modulation of gut by polyphenols can prevent and treat AD and other neurodegenerative diseases. This review summarizes the role of oxidative stress, inflammation, and GM in AD. Further, it provides an overview on the ability of polyphenols to modulate gut dysbiosis, oxidative stress, and inflammation against AD.

## 1. Introduction

The imbalance between oxidants and antioxidants in living organisms that occurs due to the inappropriate functioning of the antioxidant system or excess level of reactive oxygen species (ROS)/reactive nitrogen species (RNS) is known as oxidative stress [1]. On the other hand, inflammation is a complex set of interactions between cells and soluble factors. It arises in any tissue as a protective and adaptive response of the innate immune system during injury to re-establish the homeostasis of damaged tissues [2,3]. The proper regulation of the inflammation mechanism is necessary to avoid uncontrolled amplification and prevent the change from the normal tissue repair toward diseases onset and collateral damage [4]. An uncontrolled generation of reactive species triggers the production of more highly reactive species (a condition of oxidative stress) and ensuing perpetuation of inflammation. The excessive reactive species can damage the structure of DNA, lipids, and protein and can lead to aging [5]. In addition, it can promote cell death that activates necrosis, apoptosis, and extracellular matrix breakdown and releases various intracellular and extracellular factors to hyperactivate the inflammatory cascade, resulting in increased oxidative stress and free radical production in a vicious circle [6]. Both oxidative stress and inflammation give rise to the etiopathogenesis of many chronic disorders including cancer, diabetes, metabolic syndromes, and cardiovascular and neurodegenerative diseases [7]. However, under normal physiological conditions, free radicals and inflammation are important for the prevention of chronic degenerative diseases and the maintenance of human well-being. In addition, ROS and RNS take part in the regulation of many molecular pathways such as differentiation, metabolism, survival, proliferation, and iron homeostasis [4,5].

Human intestines comprise an intricate ecological colony of microorganisms known as gut microbiota (GM), and approximately 100 trillion microorganisms live in the human gut [8]. Recently, research has been shifted from the diversity and abundance of GM to their functional aspects. Shabbir et al. [9] stated that GM take part in metabolic, neural, immune, and defence mechanisms and have an impact on the host in health and disease. Moreover, GM ferment resistant starch and dietary fibres, releasing short-chain fatty acids (SCFA: acetate, butyrate, and propionate), gamma-aminobutyric acid, serotonin, dopamine, glutamate, and acetylcholine. The impairment of GM composition, known as dysbiosis, can lead to several metabolic disorders such as ulcerative colitis, type 2 diabetes, obesity, colorectal cancer, and cardiometabolic and metabolic liver disease. Other than metabolic diseases, dysbiosis also contributes to neurological disorders, including anxiety, bipolar disorder, depression, obsessive-compulsive disorder, epilepsy, and Parkinson’s and Alzheimer’s disease (AD) through the microbiota–gut–brain axis (MGBX). However, the actual relationship and mechanism between gut dysbiosis and neurodegeneration is elusive [10].

Studies are revealing that complex interactions between GM influence oxidative stress and inflammation and resists counter-regulatory mechanisms of antioxidants. Nutritional interventions can be an effective approach to treat gut dysbiosis. Additionally, dietary polyphenols and their metabolites (via metabolism of GM) may regulate the oxidative and inflammation state of the central nervous system and can be potent agents against AD and other neurodegenerative diseases. The current paper offers an overview on the role of oxidative stress, inflammation, and GM in the pathogenesis of neurodegenerative diseases, especially AD. Additionally, the potential implications of polyphenols on GM modulation to reduce oxidative stress and inflammation to prevent and treat AD are addressed.

## 2. Inflammation and Oxidative Stress

Inflammation and oxidative stress are closely associated in the pathophysiological events where redox homeostasis (endogenous capacity of cells to deal with challenges that generate electrophiles [10] perpetually) is disrupted due to the imbalance of oxidants and reductants [11]. The leading factors that enhance chronic inflammation are the uncontrolled production of pro-inflammatory cytokines, oxidative stress, chronic infections, and alterations in the metabolism of adipose tissues. The NADPH oxidases (NOXs) and mitochondria are the primary cellular sources of ROS throughout the mitochondrial electron transport chain. Moreover, Complexes I and III of the electron transport chain are the main source of ROS production in mitochondria [12]. Parra-Ortiz et al. [13] stated that oxidative stress instigates several modifications in lipids that generate oxidized- specific products (e.g., oxidized low-density lipoprotein or cholesteryl-esters that stimulate macrophages via toll-like receptor-4 (TLR4) and spleen tyrosine kinase) that excite inflammation and induce immune responses [14]. Further, generation of ROS in adipocytes perpetuates chronic inflammation and stimulates pro-inflammatory adipokines in the target tissue [13]. Additionally, modulation of macrophages activities due to the bioenergetics and metabolic alteration increase phospholipid oxidation in tissues that leads to the modification of membrane properties and stimulate inflammation [15]. Moreover, secretions from activated macrophages such as interleukin (IL)-6, tumor necrosis factor-α (TNF-α), and other pro-inflammatory molecules (such as NO, NO synthase, cyclooxygenase-2, and ROS) can damage DNA via oxidation [16].

**Figure 1:**
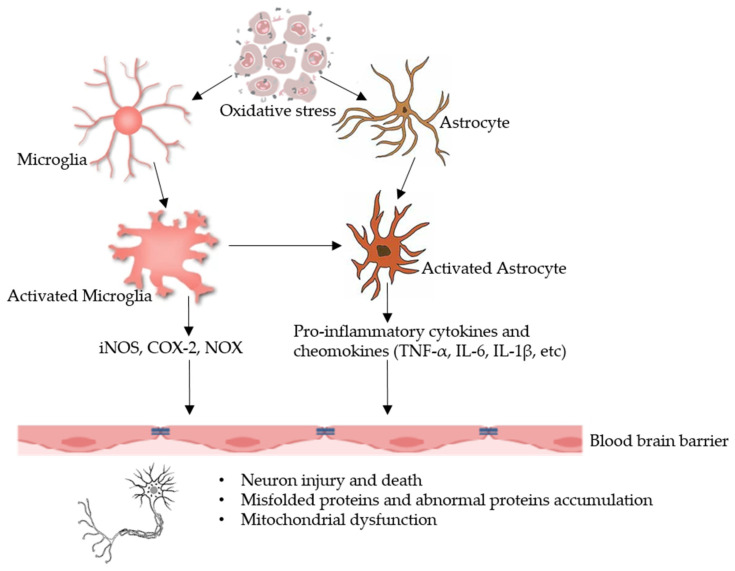
The oxidative stress leads to neuroinflammation and neurodegenration. In homeostatic conditions, astrocytes release antioxidants by degrading reactive oxygen species, uptake and metabolism of neurotransmitters, provide energy and neurotrophin. In pathological conditions, astrocytes could be activated via stimulation from activated microglia. Therefore, high levels of oxidative stress activate signalling pathways that activate microglia and astrocyte (major glial inflammatory characters). Pro-inflammatory factors secreted by glial cells induce a neuroinflammatory response that disrupts the blood brain barrier’s integrity and infiltrates into the brain, secreting factors that lead to neurodegeneration, in which the most characteristic feature is neuron injury and death. iNOS: inducible nitric oxide synthase, COX-2: cyclooxigenase-2, NOX: NADPH oxidase, IL: interleukin, TNF-α: tumor necrosis factor alpha.

Regarding molecular mechanisms, Battino et al. [7] reported that ROS activate redox-sensitive transcription factors, activator protein-1, their up-regulating kinases (especially posphoinositide 3-Kinase, extracellular signal-regulated kinases, c-Jun N-terminal kinase, and mitogen-activated protein kinases), and nuclear factor kappa-light-chain-enhancer of activated B cells (NF-κB) have a significant contribution in the pro-inflammatory responses. Studies have been reported that NF-κB, chemokines, and pro-inflammatory cytokines (e.g., IL-1β, IL-6, IL-2, IL-12, and TNF-α) recruit the neutrophils and the macrophages to the inflammation site, reinforcing the formation of oxidative species by neutrophils and macrophages which lead to the inflammation [17]. Moreover, Raucci et al. [6] and Shah et al. [18] stated that endoplasmic reticulum and mitochondrial dysfunction might be due to the excessive production of ROS that activates necrosis and apoptosis. Due to the release of high mobility group box-1 (HMGB1) through various receptors of the TLR4-dependent pathway, the necrotic tissues are responsible for the inflammation. HMGB1 as a representative damage associated molecular pattern (DAMP) protein has been documented to be involved in inflammatory diseases related to brain including stroke, epilepsy, traumatic brain injury, and hypoxic-ischemic brain injury [19,20]. Hatayama and Stonestreet et al. [19] revealed that HMGB1 translocate in damaged neurons from nucleus to cytoplasm and is released to extracellular as DAMP. They bind to receptors such as TLR4 or receptors for advanced glycation end products on astrocytes and activate them, releasing ROS, pro-inflammatory cytokines, matrix metalloproteinases, and chemokines, resulting in endothelial activation neutrophil attraction and damaging the blood-brain barrier (BBB). Neutrophils in blood vessels of the brain are activated and migrate to brain parenchyma through the damaged BBB. The activated neurological cells and migrated neutrophils release ROS, NO, and pro-inflammatory cytokines that lead to neural cell death. Figure 1 represents the role of oxidative stress is neuroinflammation and neurodegenration. 

Furthermore, HMGB1 with extracellular ATP, phagocytosis, NOX, Cathepsin B, and phagolysosomes disruption activate nucleotide-binding oligomerization domain leucine-rich repeat containing protein 3 (NLRP3) inflammasome, which enhances ROS production, thioredoxin, thioredoxin-interacting protein, and spark inflammasome activating signals that increase the agglomeration of inflammasome. The NLRP3 inflammasome also contributes to systematic inflammation and increases age-related diseases (especially neurodegenerative diseases) [21,22].

## 3. GM, Oxidative Stress and Inflammation

GM play several roles in the host, such as immune responses (as independent production of IgA antibodies, induction of T cell-dependent, promotion of IL-10, and mucosal Th17 cell response from intestinal macrophages), protection against pathogen colonization, and intestinal epithelial barrier protection. Among other functions of the GM, the production/regulation of oxidative stress is the most interesting one. It has been reported that the epithelial lining of the gut and other cell types in the presence of microbiota generate ROS. Additionally, intestinal tissues, commensal anaerobes, and leukocytes are a rich source of NO (the neurotransmitter of the non-cholinergic and non-adrenergic nervous system that exerts a neuroprotective function). Gut bifidobacteria and lactobacilli convert nitrite and nitrate in NO and increase the release of NO by host epithelial cells [23]. In addition, gut bacilli and streptomycetes produce NO via NO synthetase from L-arginine. Aberrant production of NO generates ROS associated with cellular damage, neuroinflammation, neurodegenerative disorders, and axonal degeneration [24]. Moreover, *Salmonella*, *E. coli*, and other bacteria break sulphur amino acids and produce hydrogen sulphide in the gastrointestinal tract (GIT). Higher levels of hydrogen sulphide inhibit cyclooxygenase activity, shift the metabolism towards glycolysis, increase lactate, decrease ATP production, and decrease mitochondrial oxygen consumption and overexpression of pro-inflammatory effects [25]. Other than that, He et al. [26] disclosed that trimethylamine N-oxide (TMAO; GM metabolite) is involved in oxidative stress and associated with aging and exhibited increased plasma levels of monocyte chemoattractant protein-1, IL-1β, and TNF-α, along with higher and lower plasma concentration of malondialdehyde and glutathione peroxidase/superoxide dismutase activities (implying oxidative stress). Loffredo et al. [27] and Kesika et al. [28] revealed that gram-negative bacteria (such as *E. coli* and *Shigella*) increase the production of amyloids and lipopolysaccharides (LPS) that induce local systematic inflammation and give rise to dysfunction in the permeability of GIT and BBB function during dysbiosis. GM dysbiosis increases pro-inflammatory bacteria such as *Escerchia*/*Shigella*, *Verrucomicrobia*, *Pseudomonas aeruginosa* and *Proteobacteria* and decrease the anti-inflammatory bacteria such as *Bifidobacterium*, *Bacteroides fragilis*, *Eubacterium hallii*, *Eubacterium rectale*, *Bacillus fragilis*, and *Faecalibacterium prausnitzii* that promote inflammation and contribute to neurodegenration [29]. Table 1 represents the neurodegenerative diseases induced by oxidative stress and inflammation due to gut dysbiosis.

## 4. Alzheimer’s Disease

Dementia is a general term for loss of memory, thinking ability, language, judgement, and behaviour that can deteriorate daily life activities [44]. According to Alzheimer’s Disease International, someone in the world develops dementia in every 3 s. About 50 million people have dementia globally, and this figure is expected to double in the next 20 years. Low and middle-income countries suffer the most and have around 60% of cases, which is supposed to increase (71%) by 2050 [45]. AD is the most progressive disease of the brain, comprising about 60–80% of cases of dementia and posing difficulties for families and society and a severe burden on the economy [46]. People suffering from AD may have difficulty in remembering names and recent conversions, and can have anxiety or depression in the early stages. The conditions continue to worsen over the years, leading to confusion, behavioral changes, disorientation, and ultimately facing problems in speaking, walking, swallowing, and needing extensive care [47]. The development of amyloid-beta (Aβ) plaques (Aβ-oligomers and Aβ peptides), neurofibrillary tangles, oxidative stress, neuroinflammation in the nerve cells, mitochondrial dysfunction, and insulin resistance are the hallmarks of AD [46,48,49].

### 4.1. Microbiota-Gut-Brain Axis and AD

**Figure 2:**
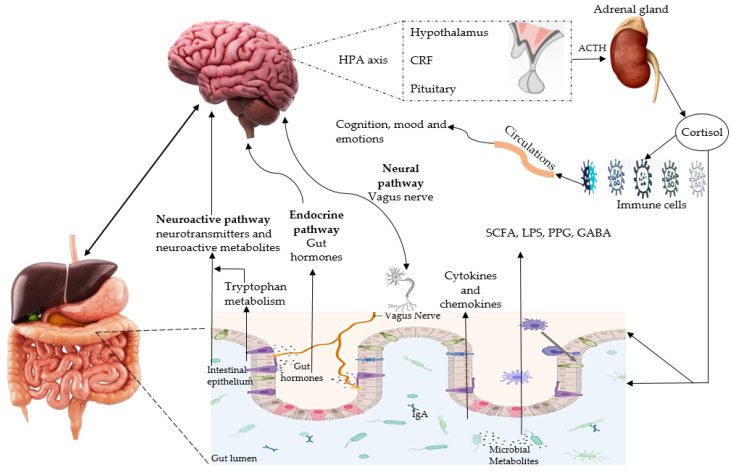
The microbiota-gut-brain axis is the bidirectional pathway between intestinal microbiota, the gut, and the central nervous system. It can be modulated by gut microbiota through endocrine (cortisol), neural (enteric and vagus nervous system), and immune (cytokines) systems. Microbial metabolites (LPS, GABA, SCFA, and PPG) and other neurotransmitters also participate in GM modulation. Gut dysbiosis can alter the tryptophan levels, hormones, SCFA, immune system, and gut permeability. Furthermore, release of cytokines and chemokines contribute to neuroinflammation and activate HPA axis (affecting gut permeability, barrier function, and immune cells through the secretion of cortisol). HPA axis: hypothalamic-pituitary-adrenal axis, ACTH: adrenocorticotropic hormone, CRF: corticotropin-releasing factor, LPS: lipopolysaccharides, GABA: y-aminobutyric acid, PPG: peptidoglycans, SCFA: short-chain fatty acids.

More than 2000 clinical trials have targeted Aβ plaques, neurofibrillary tangles, and other biomarkers but have been failed to treat AD [50]. Thereby, recent findings claim that MGBX is the bidirectional pathway that communicates through vagal and spinal nerves between gut and brain via endocrine, immune, metabolic, and neural pathways (Figure 2) and take part in the pathophysiology of AD [51]. The pro-inflammatory cytokines and bacterial metabolites (TMAO, SCFA, amyloids, LPS, and peptidoglycans) can enter into circulation via leaky gut, and can reach the brain and contribute to brain aging and cognitive decline [52,53]. Furthermore, they can interfere with Aβ_1-40_ and Aβ_1-42_ peptide interactions and hyperphosphorylation of tau, and activate glial cells leading to neurotoxic Aβ plaque formation, neuroinflammation, and neuronal degradation [54,55].

### 4.2. Oxidative Stress, Inflammation and AD: The Role of GM

Although AD is a neurodegenerative disease, preclinical and clinical studies evidently suggest altering GM is linked with AD development. The involvement of oxidative stress in the key events to initiate neural loss is clear, but determination of the immediate role of oxidative stress in the neurodegeneration process is still elusive. Markers of lipid peroxidation and high levels of protein oxidation markers (e.g., carbonyl) have been detected in both AD animal and human studies [27,56]. In this context, eubiosis in GM composition can exhibit a positive role in the reduction of reactive species through SCFA such as butyrate, while dysbiosis may contribute to systematic inflammation, activation of microglia, and BBB damage [46]. Moreover, trimethylamine is metabolized by GM, then conveyed to the liver and broken into TMAO upon oxygenation, and has been found in the cerebrospinal fluid of AD and mildly cognitively impaired (MCI) patients [53]. Additionally, Botchway and colleagues suspected that increased circulatory levels of TMAO can instigate overexpression of cytokines to elevate oxidative stress and endothelial function that results in AD and other neurodegenerative diseases [56]. A recent study on ADLP^APT^ mice (carry amyloid precursor protein (APP), tau, and presenilin-1, with six mutations) disclosed that daily transfer of fecal microbiota alleviated a myriad of AD-related pathological signs and features, including gliosis, Aβ accumulation, tau-pathology, and MCI. In addition to that, alteration in GM aggravated the gut permeability which resulted in systematic and intestinal inflammation [57]. Another study disclosed that inflammation-related taxa such as *Blautia*, *Desulfovibrio*, *Escherichia*-*Shigella*, and *Akkermansia* were distinctly changed in the APP/PSI transgenic mice (a chimeric mouse with human APP and a mutant human presenilin 1 [58]) [59]. Further, a clinical study by Wu et al. [40] revealed that alteration in GM composition is linked with pre-onset amnestic MCI and dementia AD. Saji et al. also found that higher levels of Enterotype I and III bacteria are associated with the occurrence of dementia [60]. Additionly, the abundance of *Bifidobacterium*, *Blautia*, *Lactobacillus*, and *Sphingomonas* was found higher than *Anaerobacterium*, *Papillibacter*, and *Odoribacter* in AD patients [61]. Furthermore, the abundance of *Firmicutes*, *Proteobacteria,* and *Tenericutes* at phylum, *Enterobacteriaceae, Coriobacteriaceae,* and *Mogibacteriaceae* at at family, *Phascolarctobacterium* and *Coprococcus* at genus levels was observed higher in the patients with MCI [62]. Nagpal et al. [63] revealed that not only gut bacteria contribute to AD markers but fungal-bacterial co-regulation networks also. The higher proportion of *Phaffomyceteceae*, *Sclerotiniaceae*, *Cystofilobasidiaceae*, *Togniniaceae* and *Trichocomaceae* families, *Botrytis*, *Cladosporium*, *Kazachstania*, and *Phaeoacremonium* genera and lower abundance of *Meyerozyma* were observed in the patients with MCI. Above-mentioned studies are corroborating GM as a unique factor that has the potential to affect cognitive health and can contribute to AD. Thus, diet or specific bioactive components that have the ability to modulate GM can act as potential therapeutics in MCI and AD.

## 5. Polyphenols

**Figure 3:**
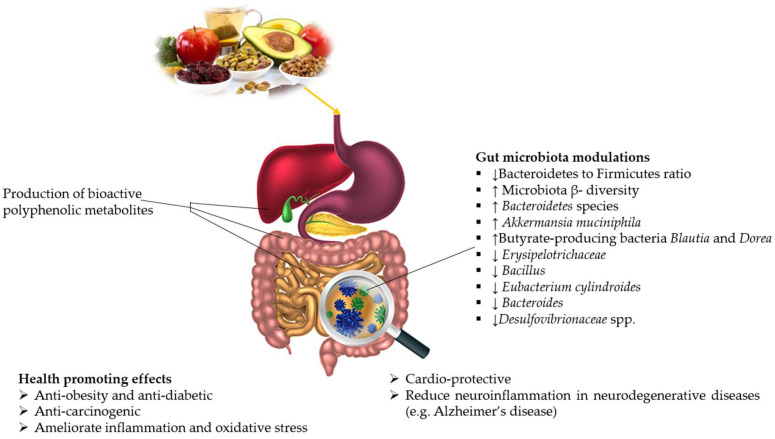
Potential health-promoting effects of dietary polyphenols and their role in gut microbiota modulation. ↑increase, ↓decrease.

Dietary polyphenols) are a group of phytochemicals that are naturally present in fruits and vegetables with potential health-promoting effects (e.g., anti-inflammatory, antioxidant, and anti-mutagenic [64,65]). Polyphenols exist in the following forms, (1) free form (such as aglycones), (2) polymers or oligomers (i.e., macromolecules), and (3) derivatives (e.g., glycosylated aglycones, acylated, or esterified). They are classified as either flavonoid (anthocyanins, flavones, flavonols, flavanones, flavanols, and isoflavones) or non-flavonoid (stilbenes, lignans, tannins, phenolic acids, and hydroxycinnamic acids) [66]. Polyphenols exert positive effects and have a broad spectrum of biological activities against many human diseases such as type-2 diabetes mellitus, cancer, cardio-metabolic diseases, and neurodegenerative diseases, as well as having potential to modulate gut dysbiosis (Figure 3) [67]. However, due to the extensive metabolism by phase-I and II enzymatic reactions and poor absorption, their bioactivity on targeted organs is significantly less. Moreover, their transformation into another chemical structure before reaching the site of action may affect their health benefits [68]. Additionally, structural stability, the impact of food matrices, solubility, interaction with GM, etc., also affect the bioavailability of polyphenols. To overcome the bioavailability issues and utilization of the beneficial properties of polyphenols, different techniques can be used, such as nanoencapsulation, microencapsulation, fermentation, or germination [8,69]. 

### 5.1. Anti-Oxidative Properties of Polyphenols

In the human diet, the most abundant antioxidants are dietary polyphenols. They can neutralize free radicals through transferring electrons/hydrogen atoms and decrease cell apoptosis via modulation of mitochondrial dysfunction. Further, they can reduce the production of hydroxyl radicals (metal-dependent) along with the chelation mechanism and instigate the nuclear factor erythroid 2-related factor 2 by inducing endogenous antioxidant enzymes [70]. They can scavenge expression of genes, ROS and RNS and activate redox-responsible transcription factors to modulate coding antioxidants, pro-survival neurotrophic factors, and anti-apoptotic Bcl-2 protein family. In addition, they can modulate the mitochondrial apoptosis system in promoting or preventing ways and can regulate mitochondrial biogenesis, autophagic degradation, and dynamics (fission and fusion) [71]. The ability of polyphenols to scavenge radicals primarily depends on the position and number of the OH groups connected with the aromatic rings [72]. In addition to OH groups, polyphenols with two or more groups –NR_2_, –PO_3_H_2_, –COOH, –O–, –SH, C=O, and –S– groups can enhance the chelation of metal ions [73]. For instance, the SH-SY5Y cells were pre-treated with butein, scopoletin, and isoliquiritigenin that protected the cell death induced by H_2_O_2_ and decreased ROS and apoptotic cells [74]. Table 2 summarizes the effects of different polyphenols on oxidative stress and inflammation (biomarkers).

### 5.2. Anti-Inflammatory Properties of Polyphenols

Many studies have stated that an increased level of pro-inflammatory molecules can act as aging indicators. However, the particular mechanisms to relate age-related diseases with inflammation and the reason why old age people are vulnerable to inflammation are still elusive [70]. As an anti-inflammatory agent, polyphenols (such as galangin, luteolin, quercetin, and epigallocatechin-3-gallate) can modulate or suppress the NF-κB activation pathway at different steps that entirely depends on the chemical structure of polyphenols [75]. Further, sirtuins 1 (family of mono-ADP-ribosyltransferase and NAD+-dependent deacylase) can inhibit and deacetylate transcription of p65 subunit of NF-κB at lysine 310 and as a result, attenuate NF-κB induced inflammatory signalling transductions. Polyphenols including quercetin [76], caffeic acid phenylethyl ester [77], hydroxycinnamic acids [78], and ferulic acid [79] activated sirtuins 1 in different study models and identified to protect against the senescence-associated secretory phenotype via NF-κB pathway inhibition. Other than NF-κB, polyphenols can also modulate the NLRP3 inflammasome, e.g., apigenin (flavone class) decreased the LPS-induced IL-6 and IL-1β production via inhibition of caspase-1 activation by interfering with the NLRP3 inflammasome assembly in mouse J774A.1 macrophages [80]. Rutin [81], quercetin [82], and anthocyanins [83] in rats or cultured cells suppressed NLRP3 inflammasome activation that restricted the related inflammatory pathways (Table 2).

**Table 2:** Summary of the effects of polyphenols on oxidative stress and inflammation (biomarkers).

Polyphenols	Study	Findings	Reference
Anthocyanins	Mouse microglial cells	↓IL-1β, TNF-α, and NO release, NF-κB nuclear translocation, COX-2 and iNOS expressions.	[84]
	Human	↓IL-6, IL-18, and TNF-α	[85]
Quercetin	Mouse BV2 microglial cells and mice	↓Oxygen glucose deprivation induced expression of inflammatory factors and TLR4/MyD88/NF-κB signalling. Ameliorated cognitive, cerebral infarct volume and motor function in mice.	[86]
	Wistar rats	↑Activity of enzymatic antioxidants and sirtuin 1, ↓NF-κB and IL-1β levels, increased IL-10 and modulated AMPK/SIRT1/NF-κB signaling pathway.	[87]
Resveratrol	SH-SY5Y neuronal cells	↓TNF-α, IL-1β, mitochondrial, and cytosolic ROS, improved the intracellular Ca^2+^ responses and mitochondrial function.	[88]
Curcumin	Sprague-Dawley rats	↓iNOS, COX-2 expression and inflammatory factor	[89]
Epigallocatechin Gallate	SPF Wistar rats	↓Acetyl-CoA carboxylase, NF-κB, and free fatty acid synthase and ↑fatty acid binding protein-1, carnitine palmitoyltransferase II and sirtuin 1.	[90]
	WI-38 cells	↑Antioxidant enzymes, superoxide dismutase 1 and 2 and ↓IL-32 and TNF-α expression.	[91]
Luteolin	Wistar rats	↓ Oxidative stress parameters, levels of NF-κB, malondialdehyde, and hydrogen peroxide and ↑glutathione S-transferase.	[92]
Kaempferol	C57 BL/6J mice	↓TNF-α and IL-6, and the activation of NF-κB and ↑NRF2/HO-1 signaling pathway and level antioxidants	[93]
Myricetin	Wistar rats	↓Markers of inflammation such as NF-κB, IL-6, TNF-α, and NRF2, ↑xanthine oxidase activity and phase-II detoxifying enzyme activity and ameliorated lipid peroxidation	[94]
Green Tea polyphenols	C57BL/6 mice	↓NLRP3 inflammasome expression, NRF2 pathways, hepatic inflammatory damage and immunological reaction	[95]
Grape Seed Extract	Human colorectal adenocarcinoma cell line Caco-2	↓Pro-inflammatory cytokine gene expression, intracellular ROS and mitochondrial superoxide production, ↑anti-inflammatory cytokines, and mitochondrial membrane potential.	[96]

↑: Higher/increased, ↓: Lower/decreased, NRF2: Nuclear factor-erythroid factor 2-related factor 2, NF-κB: nuclear factor kappa-light-chain-enhancer of activated B cells, IL: interleukin, TNF-α: tumor necrosis factor-α, NO: nitric oxide, iNOS: inducible nitric oxide synthase, ROS: reactive oxygen species, COX-2: cyclooxygenase-2, NLRP3: Nod-like receptor family, pyrin domain containing 3.

### 5.3. GM and Polyphenols

The relationship between GM and polyphenols is bidirectional as GM bio-transform polyphenols and polyphenols modulate GM. Very low (5–10%) absorption of polyphenols takes place in the small intestine while 90–95% absorption occurs in the large intestine, but bio-transformation of polyphenols in the body is dependent on the GM composition and the structure of polyphenols [8]. Lactase-phlorizin hydrolase hydrolyses the free and simple polyphenols in the small intestine, and the resulting aglycones enter the enterocyte by passive diffusion. Recycled aglycones and the polyphenols gather in the colon, where GM degrade them and facilitate absorptivity [97]. For instance, the sugar moiety of quercetin that intestinal β-glucosidases cannot hydrolyse, but GM (e.g., *Enterococcus*, *Blautia*, and *Bacteroides*) deglycosylation can, yields quercetin aglycon. Strains of *Bacteroides*, *Clostridium perfringens*, *fragilis*, *Escherichia coli*, *Enterococcus gilvus*, *Lactobacillus acidophilus*, *Streptococcus S-2*, and *Weissella confusa* can transform quercetin and other polyphenols into bioavailable metabolites [98]. Furthermore, *Clostridium saccbarogumia* and *Eubacterium ramulus* can catalyse cyaniding-3-O-glucoside into DHBA, THBAld and other products [99]. On the other hand, Wu and colleagues [70] stated that polyphenols not only act as classic prebiotics to enhance beneficial bacteria (for example *Akkermansia*, *Bifidobacterium*, *Christensenellaceae*, *Lactobacillus*, and *Verrucomicrobia*) but also inhibit pathogenic bacteria. Further, Peng et al. [100] documented that long-term consumption of anthocyanins can increase the growth of SCFA-producing bacteria such as *Barnesiella*, *Faecalibacterium*, *Odoribacter*, *Prausnitzii*, *Ruminococcaceae*, and *Roseburia*. Moreover, the consumption of neohesperidin, resveratrol combined with curcumin, green, oolong, and black tea can significantly restrain the growth of pathogenic bacteria (e.g., *Clostridiumm*, *Prevotella*, *Proteobacteria*, and *Desulfovibrionaceae*) [101,102,103]. Another study by Li et al. [104] documented that the ratio of *Firmicutes* to *Bacteroidetes* (positively correlated with many diseases) was increased after the feeding of tea polyphenols in canines. Liu et al. [105] declared that epigallocatechin-3-gallate treatment stimulated the abundance of beneficial bacteria such as *Bacteroides*, *Bifidobacterium*, and *Christensenellaceae* and inhibited pathogenic bacteria including *Bilophila*, *Enterobacteriaceae*, and *Fusobacterium varium*. Above-mentioned studies are suggesting that GM modulation by dietary polyphenols can affect MGBX and can be used as nutraceutical to treat AD and other neurodegenerative diseases.

### 5.4. Polyphenols and AD

Polyphenols have anti-oxidative and anti-inflammatory properties that abrogate ROS and RNS and sequester the production of Aβ plaques (Aβ-oligomers and Aβ peptides) and tau protein hyperphophorylation to prevent the development of neurofibrillary tangles [106]. Moreover, polyphenols (e.g., hesperidin, neohesperidin, hesperetin, and citrus flavanones) restrict neuronal disintegration by interacting with major signal transduction pathways, cerebral vasculature, and the BBB [106,107]. In addition, quercetin (RVG29-nanoparticles) showed permeability across the BBB and inhibited Aβ aggregation in thioflavin T binding assay [108]. Anthocyanins were found in the cerebellum and cortex of the mice that significantly reduced the loss of neuronal cells and memory impairment [109]. In addition, curcumin and its derivatives can pass the BBB and have neuroprotective effects against mitochondrial dysfunction, damage, and nitrosative stress [46]. Moreover, resveratrol significantly decreased the Aβ_-42_ peptide toxicity toward SH-SY5Y cells that resulted in the cleavage of Aβ_1-42_ peptides into smaller fragments [110]. Metabolism of flavan-3-ols by GM result in various arylvaleric acid, and aryl-γ-valerolactone derivatives that can selectively detoxify Aβ oligomers and prevent AD symptoms in mice [111]. Secondary metabolites of valerolactones such as hydroxybenzoic acid, (hydroxyaryl)valeric acid, (hydroxyaryl)propanoic acid, (hydroxyaryl)cinnamic acid, and (hydroxyaryl)acetic acid derivatives have more permeability across the BBB to reduce neuroinflammation and are comparatively more bioavailable than the dietary flavonoids or flavanoids [112]. Moreover, metabolites of epicatechin, 5-(4′-Hydroxyphenyl)-γ-valerolactone-3′-O-glucuronide and 5-(4′-Hydroxyphenyl)-γ-valerolactone-3′-sulfate modulate cellular pathways such as focal adhesion, cell adhesion, signalling pathways, and cytoskeleton organization to preserve brain vascular endothelial cell integrity [113]. Therefore, polyphenols, including those derived from GM metabolism, can be effective therapeutics to treat neurodegenerative diseases such as AD. Several in vivo and in vitro examples are illustrated in Table 3.

## 6. Research Limitations

Although polyphenols have demonstrated anti-oxidative and anti-inflammatory properties in vitro and in vivo animal studies, there is still inconclusive evidence regarding their effects in human studies. Moreover, the possible interaction between cognitive function, GM composition, and polyphenols has been studied well in animal studies. However, clinical studies have been carried out with a small number of samples that are lacking the comprehensive profiling in GM composition and functionality. Besides, the inconsistency in the results of GM has been observed, which may be due to the difference in the species and nutrients status of animals, treatment time and method, and composition and concentration of polyphenols in the diet. The health benefits of the polyphenols are derived from the GM metabolites that are bioavailable to the host and the interplay between the reshaping of GM, whereas, the mechanism of the GM reshaping is still elusive and may occur either by the parent compounds alone or microbial-derived polyphenolic metabolites. Thus, accurate microbiome studies are needed for future clinical diet interventions [10]. Additionally, more studies are required to check the appropriate concentration of polyphenols for their beneficial and adverse effects [126]. Furthermore, the implementation of artificial intelligence, machine learning algorithms, and use of large datasets are required to understand the complex network of interactions amongst the polyphenols, GM, and host metabolome.

## 7. Conclusions

There is a close interrelationship between oxidative stress and inflammatory pathways. Each may appear before or after the other and take part in the progression of several chronic diseases [14]. Moreover, gut dysbiosis has been reported to exert regulatory functions on oxidative stress and inflammation and play a role in neurodegenerative diseases, especially AD via MGBX. A balanced diet enriched in antioxidants such as polyphenols can be helpful in maintaining gut homeostasis (eubiosis) by counteracting oxidative stress and inflammation. In this study, we explored the possible role of polyphenols in scavenging free radical species, inhibiting the formation of pro-inflammatory cytokines, increasing anti-inflammatory cytokines, and maintaining gut dysbiosis. Despite the mentioned benefits, they have low bioavailability due to their complex absorption and metabolic process to enter into the bloodstream and succeeding to the target location. Thus, further studies are required to develop methods to improve the stability, permeability, and solubility of dietary polyphenols for their usage in nutraceutical and pharmaceutical applications to develop an efficient approach for preventing and treating neurodegenerative diseases. 

## Figures and Tables

**Table 1:** Neurodegenerative diseases influenced by the change of GM (induced by oxidative stress and inflammation).

Disease	Study	Change in GM	Findings	Reference
Major Depressive Disorder	Human (*n* = 36)	Phylum Firmicutes and Actinobacteria were overrepresented, ↑*Bifidobacterium* and *Blautia* at the genus level.	Sucrose, starch and pentose phosphate metabolism were important pathways for depression via GM functions.	[30]
	Human (*n* = 90)	*Paraprevotella* showed positive correlation while *Clostridia*, *Clostridiales*, *Firmicutes*, and the RF32 order negatively correlated with depression.	Integrity intestinal and inflammation markers were linked with the response to treat the MDD.	[31]
Anxiety	Human (*n* = 9)	↑*Fusobacterium*, *Ruminococcus gnavus*, and *Escherichia*/*Shigella*↓Microbial richness and diversity.	Enhanced gut permeability and the abundance of pro-inflammatory bacteria linked with neuroinflammation.	[32]
	Human (*n* = 36)	↑Bacteroidaceae, Bacteroides, Betaproteobacteriales, Burkholderiaceae, Tyzzerella 3, *Escherichia*/*Shigella*, Hungatella, Enterobacteriales, and Enterobacteriaceae.	The abundance of *Ruminococcaceae*_UCG-014, *Eubacterium_coprostanoligenes group*, and *Prevotella*_9 was negatively associated with anxiety severity and positively with anxiety reduction, whereas *Escherichia*/*Shigella* and *Bacteroides* was positively correlated with anxiety severity.	[33]
Obsessive-Compulsive Disorder	Human (*n* = 43)	↓species richness, evenness, and abundance of *Anaerostipes*, *Odoribacter*, and *Oscillospira*.	C-reactive protein was increased that demonstrated mild to strong linkage with psychiatric symptomatology.	[34]
Parkinson’s Disease	Human (*n* = 40)	↑relative abundance of Ruminococcaceae and Rikenellaceae family and *Barnesiella*, *Alistipes*, *Odoribacter*, and *Butyricimonas* genera.	Significant enhancement in genera from the Porphyromonadaceae family and decrease in the abundance of genera *Blautia* and *Ruminococcus* was observed in PD patients with compromised cognitive ability.	[35]
	Human (*n* = 111)	↑*Firmicutes* enterotype ↓*Prevotella* enterotype	Increased intestinal inflammatory responses, reduced SCFA level, and shifts in microbiota-host interactions between earlier PD onset.	[36]
Schizophrenia	Human (*n* = 194)	↑*Bacteroidetes*, ↓Firmicutes and Actinobacteria	Metabolic disturbance (levels of glucose, low-density lipid-cholesterol, high-density lipid-cholesterol, triglyceride, and homeostasis model assessment of insulin resistance) was observed in the patients.	[37]
Bipolar Disorder	Human (*n* = 46)	↓microbiota diversity, ↑*Clostridiaceae* and *Collinsella*	Differences in GM colonization may modulate metabolic and metabolomic alterations and other biological processes such as inflammation.	[38]
	Human (*n* = 53)	↓*Bacteroidetes*, ↑Actinobacteria and Firmicutes	Change in GM can be a potential biomarker.	[39]
Dementia	Human (*n* = 77)	↓*Clostridia*, *Clostridiales Ruminococcaceae*, Firmicutes, and *Ruminococcus*	Decrease in indole-3-pyruvic acid and SCFA producing bacteria as a signature for discrimination and prediction of dementia.	[40]
Epilepsy	Human (*n* = 40)	↑*Delftia*, *Campylobacter*, *Lautropia*, *Haemophilus*, and *Neisseria* genera among Proteobacteria phylum and *Leptotrichia* and *Fusobacterium* genera among Fusobacteria phylum	Inflammation and autoimmune mechanisms due to the taxonomic drift and differences in the intestinal microbiota have a role in the etiology of epilepsy.	[41]
	Human (*n* = 44)	↑*Ruminococcus_g2* and *Bacteroides finegoldii* in drug-resistant group, *Negativicutes* from *Firmicutes* in drug-resistant group and *Bifidobacterium* in all patients.	Alteration in GM can be a biomarker to evaluate and diagnose the treatment response in patients.	[42]
Huntington’s Disease	R6/2 HD mice	↑abundance of *Bacteroidetes* and ↓*Firmicutes*	Different compositions of *Bacteroides*, *Coprobacillus*, *Enterobacteriaceae, Lactobacillus*, and *Parabacteroides* were found in diseased animals.	[43]

↑: Higher/increased, ↓: Lower/decreased, PD: Parkinson’s disease, SCFA: short-chain fatty acids, MDD: Major depressive disorder, GM: gut microbiota, R6/2 HD mice: expressing exon 1 of the Huntington’s disease gene, *n*: number of total patients taken part in the study but the columns of change in GM and findings are only representing the data of diseased ones.

**Table 3:** Potential role of polyphenols in AD and related findings.

Polyphenols	Study	Findings	Reference
Curcumin	APP/PS1 double transgenic mice	Change in *Lactobacillaceae*, *Rikenellaceae*, *Prevotellaceae*, and *Bacteroidaceae* at family level, and *Bacteroides*, *Prevotella*, and *Parabacteroides* at genus level. Curcumin reduced the Aβ plaques burden and improved the cognitive abilities.	[114]
Quercetin-3-O-Glucuronide	Mice and SH-SY5Y Cells	Ameliorated tau phosphorylation, and Aβ plaques. Restored CREB and brain-derived neurotrophic factor levels in the hippocampus, and gut dysbiosis.	[115]
Quercetin	Adult male albino rats	Protected and prevented neuronal damage in the hippocampus.	[116]
RSV, QCT and API	Human SK-N-BE and SH-SY5Y cells	Reduced mitochondrial and peroxisomal dysfunction, 7KC-induced toxicity and cell death.	[117]
Luteolin	Sprague-Dawley rats	Down-regulated the expression of BASE1 and NF-κB and reduced Aβ levels in the hippocampus and cortex. Moreover, increased antioxidant potential, and suppressed inflammation and lipid peroxide production.	[118]
Palmitoylethanolamide and Luteolin	Sprague-Dawley rats	Up-regulated the gene expression of enzymes, pro-inflammatory cytokines, and reduction of mRNA levels.Moreover, inhibited the Aβ-induced astrogliosis and microgliosis.	[119]
Bilberry Anthocyanins	Sprague-Dawley rats	Enhanced the growth of *Aspergillus oryzae*, *Bacteroidales-S24-7-group*, *Bacteroides*, *Clostridiaceae-1*, *Lactobacillus*, and *Lachnospiraceae_NK4A136_group* and inhibited the growth of *Verrucomicrobia* and *Euryarchaeota* in aging rats.	[120]
	APP/PSEN1 transgenic AD mice	Down-regulated the expression of inflammatory factors, chemokine receptor CX3CR1, serum and brain LPS. Reversed the brain, kidney, and liver injury caused by AD.	[121]
Tea Polyphenols	Aging model rats	Prevented memory decline and TLR4/NF-κB inflammatory signal pathway. Besides, significantly improved the composition and diversity of intestinal microflora, shape and function of epithelium, and brain inflammation.	[122]
Epigallocatechin-3-Gallate	Sprague-Dawley rats	Decreased the tau hyperphosphorylation in hippocampus and expression of BACE1 and Aβ_1-42_ by improving the antioxidant system, learning and memory function.	[123]
Resveratrol	AD transgenic 5XFAD	Prevented memory loss and reduced the amyloid burden and tau pathology.	[124]
Berberine	Sprague-Dawley rats	Production of COX-2, TNF-α, IL-12, IL-6 and IL-1β was normalized, inhibited the production of Aβ42 and evoked the formation of antioxidant Aβ40.	[125]

AD: Alzheimer’s disease, RSV, QCT and API: Resveratrol, Quercetin, and Apigenin, BACE1: β-site amyloid precursor protein cleaving enzyme, NF-κB: nuclear factor-κB, Aβ: amyloid beta, TNF-α: tumor necrosis factor-α, IL: interleukin, LPS: lipopolysaccharide, COX-2: cyclooxygenase-2, CREB: cyclic AMP response element binding protein.

## Abbreviations

GM	Gut microbiota
AD	Alzheimer’s disease
ROS	Reactive oxygen species
RNS	Reactive nitrogen species
SCFA	Short-chain fatty acids
MGBX	Microbiota-gut-brain axis
NOXs	NADPH oxidases
NO	Nitric oxide
TLR4	Toll-like receptor-4
IL-6	Interleukin
GIT	Gastrointestinal tract
APP	Amyloid precursor protein
TNF-α	Tumor necrosis factor-α
HMGB1	High mobility group box-1
DAMP	Damage associated molecular pattern
NF-κB	Nuclear factor kappa-light-chain-enhancer of activated B cells
NLRP3	Nucleotide binding oligomerization domain leucine rich repeat containing protein 3
TMAO	Trimethylamine N-oxide
LPS	Lipopolysaccharides
Aβ	Amyloid-beta
MCI	Mild cognitive impaired
MDD	Major depressive disorder
PD	Parkinson’s disease
BBB	Blood-brain barrier
NRF2	Nuclear factor-erythroid factor 2-related factor 2
iNOS	Inducible nitric oxide synthase
COX-2	Cyclooxygenase-2
CREB	Cyclic AMP response element binding protein.
